# HPLC-ESI-MS method for C_60_ fullerene mitochondrial content quantification

**DOI:** 10.1016/j.dib.2018.06.089

**Published:** 2018-07-10

**Authors:** Anna Grebinyk, Sergii Grebinyk, Svitlana Prylutska, Uwe Ritter, Olga Matyshevska, Thomas Dandekar, Marcus Frohme

**Affiliations:** a Division Molecular Biotechnology and Functional Genomics, Technical University of Applied Sciences Wildau, Hochschulring 1, 15745 Wildau, Germany; b Dept. of Bioinformatics, Biocenter, University of Würzburg, Am Hubland, 97074 Würzburg, Germany; c Educational and Scientific Center "Institute of Biology and Medicine", Taras Shevchenko National University of Kyiv, Volodymyrska 64, 01601 Kyiv, Ukraine; d Dept. of Chemistry, Taras Shevchenko National University of Kyiv, Volodymyrska 64, 01601 Kyiv, Ukraine; e Institute of Chemistry and Biotechnology, University of Technology Ilmenau, WeimarerStraße 25 (Curiebau), 98693 Ilmenau, Germany

## Abstract

The presented dataset describes the quantification of carbon nanoparticle C_60_ fullerene accumulated in mitochondria of human leukemic cells treated with nanostructure. Firstly, the high performance liquid chromatography–electro spray ionization–mass spectrometry (HPLC-ESI-MS) method was developed for quantitative analysis of pristine C_60_ fullerene. Then, human leukemic cells were incubated with C_60_ fullerene, homogenized and subjected to the differential centrifugation to retrieve mitochondrial fraction. The C_60_ fullerene content was quantified by HPLC-ESI-MS in extracts of cellular fractions.

This data article refers to the research article “C_60_ Fullerene Accumulation in Human Leukemic Cells and Perspectives of LED-mediated Photodynamic Therapy” by Grebinyk et al. [1].

## Specifications Table

**Table:** 

Subject area	*Biology, Chemistry*
More specific subject area	*Nanoparticle uptake*
	*Liquid chromatography–mass spectrometry*
Type of data	*Table, figures with graphs*
How data was acquired	*UV-Spectrophotometer UV-1800 (Shimadzu, Kyoto, Japan)*
	*Nexera HPLC system coupled to the LCMS-8040 Tandem Quadrupole Mass Spectrometer, equipped with an Electro Spray Ionization source (Shimadzu, Kyoto, Japan)*
Data format	*Analyzed data*
Experimental factors	*Human leukemic cells CCRF-CEM incubated in the presence of 20 µM C*_*60*_*fullerene for 24 h*
Experimental features	*HPLC-ESI-MS analysis of C*_*60*_*fullerene content in human leukemic cell homogenate and cellular fractions*
Data source location	*Hochschulring 1, 15745 Wildau, Germany*
Data accessibility	*Data are available with this article*

## Value of the data

• The data article presents a novel HPLC-ESI-MS method for quantitative analysis of pristine C_60_ fullerene intracellular content.
• The experimental design combines different steps and various methodologies, starting from cell culture up to mass spectrometry analysis.
• The developed set-up could be used to quantify the content and distribution of pristine C_60_ fullerene and other carbon nanostructures in cell and its compartments.

## 1 Experimental design and data

Quantification of a nanostructure distribution within the cell enlightens its possible impact on whole cell metabolism. Herein, we present a novel high performance liquid chromatography–electro spray ionization–mass spectrometry (HPLC-ESI-MS) quantification method of intracellular content of carbon nanostructure – C_60_ fullerene. This data article refers to the recent work by Grebinyk et al. [1].

Liquid chromatography separation and mass spectrometric detection were achieved by employing the Nexera HPLC system coupled to the LCMS-8040 Tandem Quadrupole Mass Spectrometer, equipped with an ESI source (Shimadzu, Kyoto, Japan). Chromatographic separation was performed using the column Eclipse XDV-C8 150 mm × 4.6. mm, 5 μm(Agilent, Santa Clara, USA) with an isocratic mobile phase of toluene and methanol (45:55, v-v). Chromatographic conditions and optimized MS parameters are presented in Table 1. For data processing the software LabSolutions LCMS (Shimadzu, Kyoto, Japan) was used.

**Table 1:** HPLC-ESI-MS conditions for analysis of C_60_ fullerene.

Chromatographic conditions	
Column	Agilent Eclipse XDB-C8
Column temperature	40 °C
Mobile phase	methanol:toluene (45:55, v-v)
Flow rate	0.7 ml min
Run time	5 min
Injection volume	3 µl

MS conditions	
Ionization source	ESI
desolvation line temperature	250 °C
heat block temperature	400 °C
Target molecular ion	720 [M]^+^*m*/*z*
Time window	0–5 min
Dwell time	0.2 s
Interface voltage	4.5 kV
Nebulizing gas flow	3 l/min
Drying gas flow	15 l/min

**Fig. 1:**
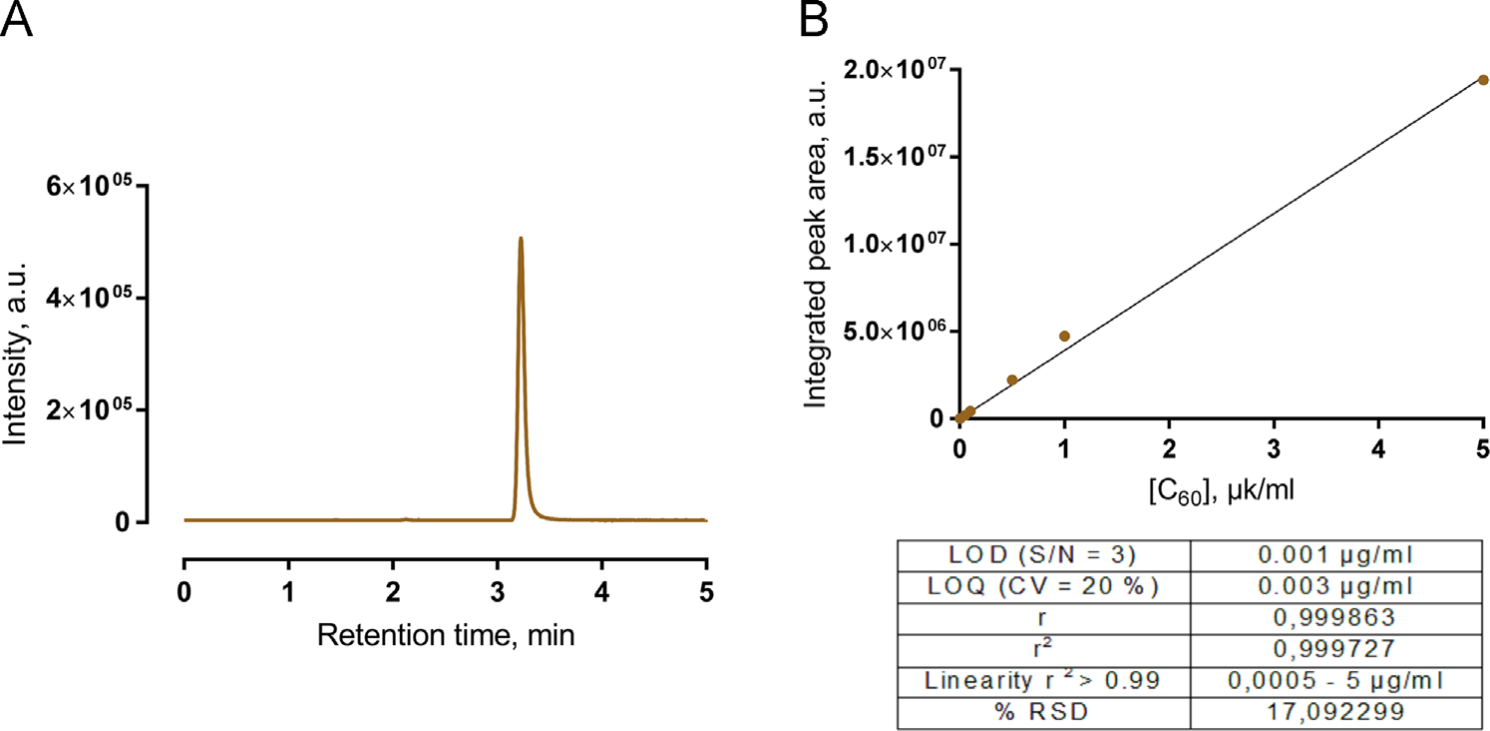
Data of the developed HPLC-ESI-MS method for C_60_ fullerene detection and quantification: **A** – representative SIM-chromatogram of C_60_ fullerene from cell extract, **B** – calibration curve with methods performance characteristics used for nanoparticle content quantification.

MS chromatograms of C_60_ fullerene (retention time = 3.21 min) were acquired using single ion monitoring (SIM) mode in positive regime with target molecular ion = 720 [M]^+^ *m*/*z* (Fig. 1A). Acquisition time was 0.2 s. Quantification was achieved using regression curves in the range 0.0005–5 µg/ml (Fig. 1B). The regression equation was “*y* = (3.86582*e* + 006)*x* + 92537”. The limit of detection (LOD) was defined according to: LOD = 3.3 × *s*/Slope, where s is the standard deviation of the regression line. The Limit of quantification (LOQ) was estimated by the serial dilution of the standard solution (*n* = 3 per dilution) and defined as the concentration at which precision was ≤ 20%. The good linearity ranges were achieved by the analysis of linear correlation coefficient for C_60_ *r* = 0.99986. All the analyses were performed in triplicate, and the peak areas were measured.

**Fig. 2:**
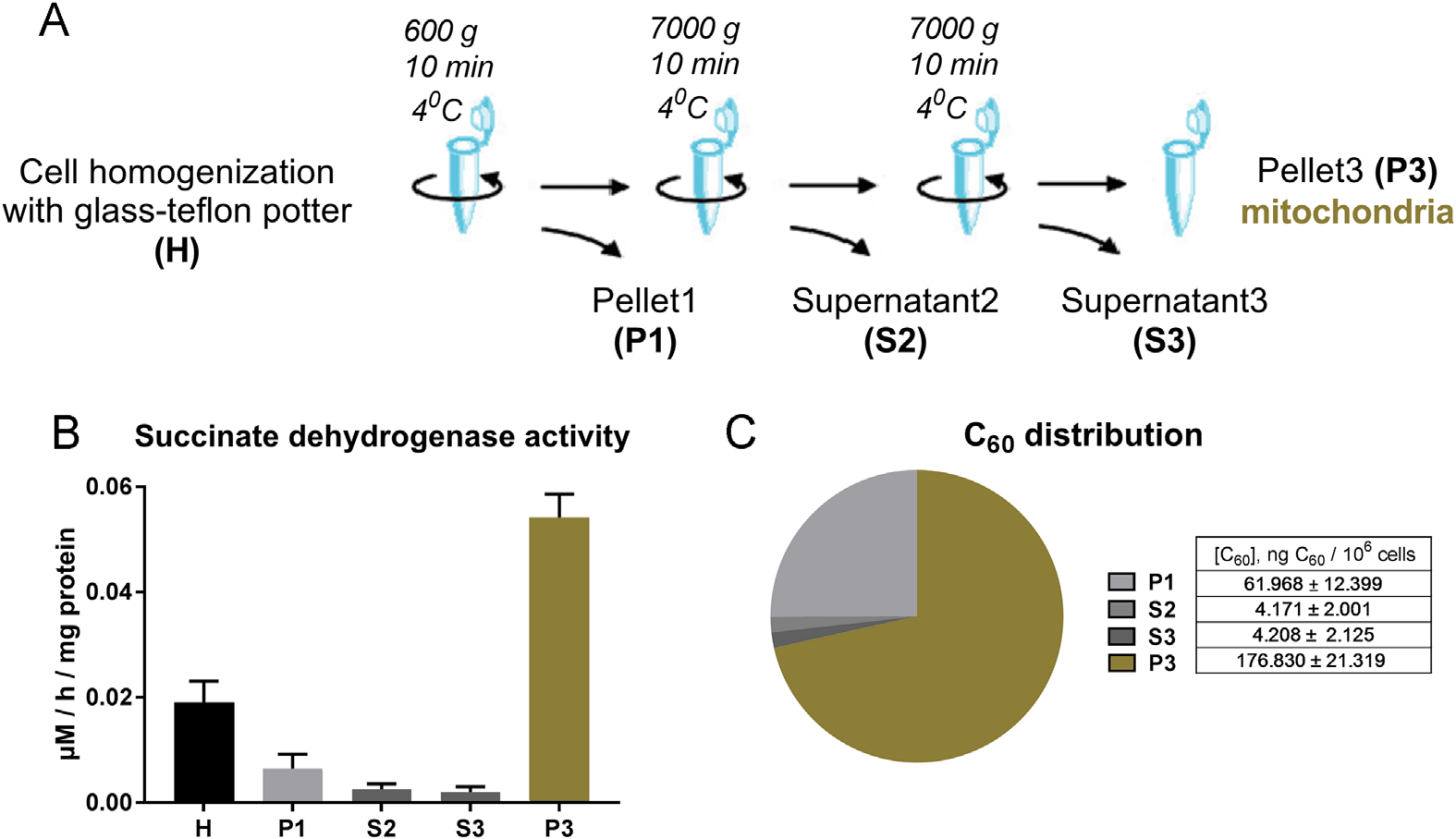
C_60_ fullerene mitochondrial content quantification: **A** – Isolation of mitochondria, **B** – Succinate-reductase activity of obtained cellular fractions, **C** – C_60_ fullerene distribution among cellular fractions.

The full experimental set-up flow combines different methods starting from cell culture up to mass spectrometry as well as obtained relative data (Fig. 2). The aim was set to quantify C_60_ fullerene content in the mitochondria of leukemic cells with above presented HPLC-ESI-MS method. For that, human leukemic cells CCRF-CEM were incubated in the presence of C_60_ fullerene for 24 h followed by homogenization and differential centrifugation. Succinate-reductase activity was used as a mitochondrial marker to test fraction enrichment and purity (Fig. 2B). Finally, C_60_ fullerene concentration was estimated in cellular fractions (Fig. 2C).

## 2 Materials and methods

### 2.1 Chemicals

RPMI 1640 liquid medium, phosphate buffered saline (PBS), Fetal Bovine Serum (FBS), Penicillin/Streptomycin and L-glutamin were obtained from Biochrom (Berlin, Germany). C_60_ fullerene, sucrose, trichloroacetic acid, Coomassie Brilliant Blue G-250, ethanol and bovine serum albumin (BSA) were obtained from Sigma-Aldrich Co. (St-Louis, USA). Toluene, methanol, 2-isopropanol, acetonitrile, tris(hydroxymethyl)aminomethane (Tris), ethylene glycol-bis(β-aminoethyl ether)-N,N,N׳,N׳-tetraacetic acid (EGTA) and phosphoric acid from Carl Roth GmbH+Co. KG (Karlsruhe, Germany) were used. 3-(N-morpholino)propanesulphonic acid (MOPS) was purchased from ICN Biomedicals Inc. (Ohio, USA). 2-(4-iodophenyl)-3-(4-nitrophenyl)-5-phenyl-2H-tetrazolium (INT) and ethyl acetate were obtained from Acros Organics (Geel, Belgium).

### 2.2 C_60_ fullerene aqueous colloid solution preparation

The pristine C_60_ fullerene aqueous colloid solution was prepared as described in [2] by C_60_ fullerene transfer from toluene to water using continuous ultrasound sonication. Obtained C_60_ colloid solution was characterized by high C_60_ fullerene concentration (2 × 10^−4^ М, purity 99%), stability and homogeneity.

### 2.3 Cell culture

The human cancer cell line of leucosis origin – CCRF-CEM (ACC 240) – was purchased from the Leibniz Institute DSMZ-German Collection of Microorganisms and Cell Cultures. Cells were maintained in RPMI 1640 medium supplemented with 10% Fetal Bovine Serum, 1% Penicillin/Streptomycin and 2 mM Glutamine, using 25 cm^2^ flasks at a 37 °C with 5% CO_2_ in a humidified incubator Binder (Tuttlingen, Germany). The number of viable cells was counted using 0.1% trypan blue staining and a Roche Cedex XS Analyzer (Basel, Switzerland).

### 2.4 C_60_ fullerene extraction

CCRF-CEM cells (2 × 10^5^/ml) were seeded in 6-well plate Sarstedt (Nümbrecht, Germany). After 24 h cells were incubated for 0–48 h in the presence of 20 µM C_60_, washed with PBS three times and transferred to the dH_2_0. The freeze-thawing cycle was repeated three times. The probes were dried at 80 °C under reduced pressure. Toluene/2-propanol (6:1, v/v) was added in the final volume 0.5 ml, the mixture was sonicated for 1 h and centrifuged (70 min, 20 238 *g*). The toluene layer was analyzed with HPLC-ESI-MS.

### 2.5 Isolation of mitochondria

CCRF-CEM cells were incubated for 24 h in the presence of 20 µM C_60_ from aqueous colloid solution and the mitochondria fraction was isolated accordingly to [3]. Cell suspension (5 × 10^6^/4 ml) was centrifuged at 600 *g* at 4 °C for 10 min, cells were resuspended in 3 ml of ice cold isolation buffer (0.01 M Tris-MOPS, 1 mM EGTA/Tris, 0.2 M sucrose, pH 7.4) and homogenized in the teflon-glass potter on ice. The homogenate was centrifuged at 600 *g* at 4 °C for 10 min. The collected supernatant (S1) was centrifuged at 7000 *g* at 4 °C for 10 min. The pellet (P2) was resuspended in 200 µl ice-cold IB and centrifuged at 7000 *g* at 4 °C for 10 min. The mitochondrial fraction obtained in pellet (P3) was used for extraction of C_60_ fullerene as well as for measurements of protein concentration [4] and succinate-reductase (SR, EC 1.3.5.1) activity as mitochondrial marker [5].

#### 2.5.1 Protein concentration assay

The protein concentration colometric assay is based on the proportional binding of the dye Coomassie to proteins, changing its color from brown to blue. Aliquots of both homogenate and all fractions after differential centrifugation were used to determine the protein concentration. After incubation with 0.01% Coomassie working solution in 4.7% ethanol and 8.5% phosphoric acid for 5 min. Protein concentration was estimated at 595 nm, using BSA as a protein standard.

#### 2.5.2 Succinate-reductase activity assay

Succinate-reductase activity colometric assay is based on the reduction of a tetrazolium salt INT to cyan farmazan. 50 µl aliquotes of homogenate as well as of fractions after differential centrifugation were shaken for 15 min at 37 °C in 0.5 ml of the working solution (0.1% INT, 50 mM sodium succinate, 25 mM sucrose in 50 mM potassiumphosphat buffer, pH 7.4). To stop the reaction the proteins were precipitated with 0.5 ml 25% trichloroacetic acid. The farmazan was extracted with 2 ml ethyl acetate. The absorption of organic colored layer was analyzed at *λ* = 492 nm with UV-Spectrophotometer UV-1800 (Shimadzu, Kyoto, Japan). The succinate-reductase activity was calculated using extinction coefficient (Δ*E*) by the following formula:

SRActivityUnits[μM/h/mgProtein]=4×ΔE/20.1/ProteinConcentration
