# Supplementary material for: HPLC-ESI-MS method for C60 fullerene mitochondrial content quantification
Source: Data Brief. 2018 Jul 10;19:2047–52. doi: 10.1016/j.dib.2018.06.089 (PMC6141383; doi:10.1016/j.dib.2018.06.089)
Supplement: Supplementary file 1 — Supplementary material. [file mmc1.docx]

**Competing Interests**

The authors declare that they have no competing interests.
